# Assessment of methods for prediction of human West Nile virus (WNV) disease from WNV-infected dead birds

**DOI:** 10.1186/1742-7622-6-4

**Published:** 2009-06-05

**Authors:** Anna Veksler, Millicent Eidson, Igor Zurbenko

**Affiliations:** 1 School of Public Health, University at Albany, One University Place, Rensselaer, New York 12144, USA; 2 Zoonoses Program, New York State Department of Health, 621 Corning Tower, Empire State Plaza, Albany, New York 12237, USA

## Abstract

**Background:**

West Nile virus (WNV) is currently the leading cause of arboviral-associated encephalitis in the U.S., and can lead to long-term neurologic sequelae. Improvements in dead bird specimen processing time, including the availability of rapid field laboratory tests, allows reassessment of the effectiveness of using WNV-positive birds in forecasting human WNV disease.

**Methods:**

Using New York State integrated WNV surveillance data from transmissions seasons in 2001–2003, this study determined which factors associated with WNV-positive dead birds are most closely associated with human disease. The study also addressed the 'delay' period between the distribution of the dead bird variable and the distribution of the human cases. In the last step, the study assessed the relative risk of contracting WNV disease for people who lived in counties with a 'signal' value of the predictor variable versus people who lived in counties with no 'signal' value of the predictor variable.

**Results:**

The variable based on WNV-positive dead birds [(Positive/Tested)*(Population/Area)] was identified as the optimum variable for predicting WNV human disease at a county level. The delay period between distribution of the variable and human cases was determined to be approximately two weeks. For all 3 years combined, the risk of becoming a WNV case for people who lived in 'exposed' counties (those with levels of the positive dead bird variable above the signal value) was about 2 times higher than the risk for people who lived in 'unexposed' counties, but risk varied by year.

**Conclusion:**

This analysis develops a new variable based on WNV-positive dead birds, [(Positive/Tested)*(Population/Area)] to be assessed in future real-time studies for forecasting the number of human cases in a county. A delay period of approximately two weeks between increases in this variable and the human case onset was identified. Several threshold 'signal' values were assessed and found effective at indicating human case risk, although specific thresholds are likely to vary by region and surveillance system differences.

## Background

West Nile virus (WNV) was first recognized in the Northeast United States in 1999 [1]. Since 1999 the virus has spread across the country, resulting in 28,943 human cases and 1130 deaths through 2008 (reported as of February 13, 2009) [2]. WNV is now found throughout the western hemisphere [3]. Infection can lead to long-term neurologic sequelae in people [4], and is currently the leading cause of arboviral-type encephalitis in the U.S. [5].

The number of human cases reported to the Centers for Disease Control and Prevention (CDC) currently varies widely in the U.S., from a low of 1 case in North Carolina, South Carolina and West Virginia in 2006 to a high of 996 cases in Idaho [6]. Some of this variation can be attributed to different human population sizes and surveillance systems for infection, with some states more aggressive at testing and reporting milder, non-neuroinvasive disease. Although most WNV exposures do not result in clinical disease and most disease is mild, with case fatality rates ranging from 3% to 15% [7] disease prevention should be a priority. Personal protective measures and mosquito control measures all have a resource cost, and thus it is important to try and determine when the risk of human infection is high or low so that individuals and governmental agencies can make appropriate decisions about prevention.

Dead bird surveillance has served as one key method for tracking WNV activity in the U.S. [8-10]. Previous studies have documented that dead crow sightings can serve as a valuable index for forecasting human cases before or without laboratory confirmation of WNV infection, because crows have had a case-fatality rate close to 100% and are reasonably easy for the public to recognize and report [11,12]. Multiple laboratory diagnostic methods may be used for serologic and virologic diagnosis of WNV, although collection, submission, processing, and testing can be lengthy [13,14]. Thus, dead crow sightings, which can be used for surveillance immediately upon reporting, provide a more immediate indicator of WNV activity than WNV-positive birds. However, more rapid processing methods are in use in many areas, including VecTest and RAMP, that can be used to provide a laboratory result from swabs taken where the bird is found [13,15]. Development of dead bird indicators using only WNV-positive birds eliminates the possibility of misclassification inherent in a dead crow sighting index, and it allows use of other species, which is particularly important in areas with few crows or other corvids (blue jays, ravens), or where WNV has reduced the crow population. Previous studies have indicated that WNV is sometimes first detected in an area in a bird species other than a corvid [16].

This study is an exploratory study focusing on determining which factors, using WNV-positive and tested dead birds, are most closely associated with the number of human WNV cases at a county level. The study also addresses the issue of "delay" between the distribution of a predictor variable based on laboratory-tested dead birds and the distribution of human disease. Previous studies have noted that the time between mosquito bites and human disease onset (incubation period) is 2–14 days [17]. This study expands upon prior analyses by considering daily distributions of the predictor variable and human disease and applying statistical methods to assess the "delay" period between these variables. Finally, this study assesses the predictive value for the risk of human disease of using a weekly county level predictor variable based on WNV-positive and tested dead birds with several signal levels.

### Analysis

#### Data

To identify measures of WNV activity in birds that might provide an indication of increased risk in humans, analyses focused on NYS integrated WNV surveillance data collected during the transmission season, defined as a 16-week period using weeks as defined for reporting by the CDC as the 26^th ^week to the 41st week for the years 2001–2003 (June 24–Oct. 13 in 2001, June 23–Oct. 12 in 2002, and June 22–Oct. 11 in 2003). This time frame was optimal for inclusion of all human disease in New York State.

The NYS integrated surveillance system includes real-time surveillance components for humans, mammals, birds, and mosquitoes. The surveillance system relies on the public to report sightings of dead birds to local health departments (LHDs). Some of the reported dead birds were collected and tissue specimens were tested for WNV infection at the NYSDOH Wadsworth Center's Arthropod-borne Disease Laboratory, according to laboratory protocols previously described [18].

For WNV surveillance in humans, healthcare providers were asked to report patients with encephalitis and aseptic meningitis to the LHDs. Thus, the NYSDOH surveillance system emphasizes reporting of human cases with neuroinvasive disease, although cases without neuroinvasive disease are not excluded. Patients were tested for WNV infection at the NYSDOH Wadsworth Center's Diagnostic Immunology Laboratory (serology), Arbovirus Laboratory (PRNT), and Viral Encephalitis Laboratory (PCR), according to laboratory protocols previously described [19].

According to the CDC case definitions, New York (excluding New York City) had six (one excluded from study) confirmed or probable human WNV cases disease in 2001, 52 (three excluded) in 2002 and 40 (three excluded) in 2003. Two counties had human WNV cases in 2001, 12 counties had human WNV cases in 2002, and ten counties reported WNV human cases in 2003. Data from 57 NYS counties were included in this study. New York City data were excluded from analysis because New York City developed its own WNV surveillance system to monitor dead bird reports, with different priorities for reporting.

These analyses were conducted with county as the unit of analysis for several reasons. The small number of human cases made further subdivision challenging. In addition, the actual location of infection for humans and birds is unknown, so county can serve as a reasonable surrogate, acknowledging the potential for misclassification and reduced statistical power if infection actually occurred in a different county. Finally, these analyses were conducted to aid in prevention and control decision-making, which occurs at the county level.

#### Variables measuring WNV activity in dead birds

To characterize the intensity of the WNV activity in birds during the mosquito season for each county, eight variables were constructed empirically from bird surveillance variables as potential candidates for a predictor variable based on dead birds tested and confirmed with WNV at the laboratory. One set of variables was constructed by standardizing the surveillance variables WNV-positive birds and tested birds by county land area, 2000 human population estimates from the U.S. Census Bureau, or by human population density. These variables were designated as A1 through A5 (Table 1). Another set of constructed variables accounted for the intensity of the bird epizootic as measured by the proportion of tested birds that were positive for WNV (A6), or proportion of tested birds that were positive for WNV, standardized by human population or population density (A7, A8).

**Table 1:** Variables constructed empirically from positive bird and tested bird surveillance variables

Surveillance variables standardized by population of county, area of county, or population density ^a^	Proportion of positive birds and proportion of positive birds standardized by population or population density
A1 = [Positive/Area]	A6 = [Positive/Tested]
A2 = [Tested/Area]	A7 = [(Positive/Tested)*Population]
A3 = [Positive/Population]	A8 = [(Positive/Tested)*(Population/Area)]
A4 = [Positive*(Population/Area)]	
A5 = [Tested*(Population/Area)]	

^a ^Positive = WNV-positive dead birds; Tested = WNV-tested dead birds; Area = county land area in square miles; Population = county human population (U. S. Census Bureau, 2000 estimates)

To determine the significant variables among the eight variables considered as potential predictor variables using WNV-positive and/or tested dead birds, a correlation matrix between each of the variables for every county with at least one human case and the human cases in each county across all three years was generated using SAS software. The variables were considered as highly correlated with human disease if p ≤ 0.05. Multiple regression was then used to determine the best model predicting the number of human disease cases using the adjusted R^2 ^statistic (adjusted for the number of parameters in the model) [20].

#### Delay period

Previous studies have noted that WNV-positive dead birds are usually found before the onset of the human infection [21,22]. Studies in the eastern U.S. found that the delay period between the first WNV-positive bird and the first human case varied from 15 days to 92 days [22]. Possible explanations for the delay include the amplification cycle between birds and mosquitoes and the transmission cycle from mosquitoes to humans through mosquito bites. The time between the exposure (mosquito bite) and onset of human disease (incubation period) is known to be two to 14 days [17]. To determine the delay period for our study, the distribution of the variable with the highest correlation with human disease for every day of the transmission period across all counties and for all three years pooled together was considered.

To obtain the best estimate of the delay period between the occurrence of WNV-positive birds and onset of human disease, we calculated the mean value of the optimal predictor dead bird variable for every day of the transmission period across all counties with human cases for all three years. The study period each year was 16 weeks (113 days). For each of the 113 days for every county with human cases we found the values of the predictor variable identified in the previous modeling for the particular day, then summarized them across all counties and divided the sum by 24, the number of counties with human cases in 2001–2003. If no birds were tested on that day, then the predictor variable was considered as zero.

To determine the delay period, two methods, non-parametric and parametric, were considered. The non-parametric two-sample Kolmogorov-Smirnov test was used to test whether the two underlying probability distributions of the predictor variable and human disease differed significantly [23]. This test was performed using SAS software with the proc npar1way, edf option. By shifting the values of our variable against the daily distribution of human cases, the best shift in days that maximized goodness of fit between two distributions was determined. The second (parametric) method was applied to smoothed daily distributions of our predictor variable and human cases. The goal of this method was to maximize correlation between two distributions over different shifts and select the shift that provides the maximum. This test was performed using SAS proc corr for every value of shift.

#### Estimation of WNV disease risk

The Cochran-Mantel-Haenszel (CMH) test was used to calculate point and interval estimates for the relative risk of becoming a human WNV case depending on the value of the predictor variable in a person's county of residence. For each week, a table was constructed comparing the week's human cases per population in counties with a "signal" value of the predictor variable (greater than a threshold pre-determined for county use based on preliminary estimates) and the human cases per population in counties with no signal. Analyses were conducted with a weekly distribution of the predictor variable, to avoid small numbers or zeroes in daily distributions. The CMH chi-square statistic was used to compare the incidence (risk) of WNV disease in signal areas with the incidence in the non-signal areas over all the weeks of study. The CMH test was performed for several threshold values of the predictor variable using SAS Proc freq to obtain the estimate of odds ratio and relative risk [SAS System for Windows V8, SAS Institute, Cary, NC, USA].

The county was included in the CMH analysis if it tested at least 10 birds during the transmission period. In 2001, only 16 counties tested at least 10 birds. In 2002, 53 counties tested at least 10 birds. In 2003, 48 counties tested at least 10 birds. All counties with human cases, except one, were retained in the analysis because they all tested at least 10 birds during the transmission period. The exception was Schuyler County with a human case in 2003 but only five birds tested. This county was excluded from analysis in 2003.

In the CMH analysis, data were included from each week with onset of human cases – in 2001, from 8/19 to 9/22 (weeks 34 to 38), in 2002, from 7/28 to 10/5 (weeks 31 to 40), and in 2003, from 8/3 to 9/27 (weeks 32 to 39). For each week, we constructed a table that compared the number of persons with disease in counties with a "signal" (the predictor variable greater than or equal to the threshold value) and the number of persons without disease (population minus disease cases) with the number of persons with disease in counties without a "signal" and the number of persons without disease. A template for the weekly tables is presented in Table 2.

**Table 2:** Sample CMH data table for assessing relative risk of becoming a human WNV case

Week 38, 2003 Threshold = defined level^a^	In counties with predictor variable^b ^≥ threshold at defined time period^c ^before case onset	In counties with predictor variable < threshold at defined time period before case onset
Number of persons with disease onset	3	2
Number of persons without disease onset	3,653,369	7,013,746

^a^Various threshold levels were defined to assess relative risk of becoming a human WNV case depending on predictor variable value in county of residence.

^b^Predictor variable determined with multiple regression modeling.

^c^Time period defined based on delay period analysis.

## Results

### Association of WNV-positive dead birds with human WNV disease

The full correlation matrix of the number of human cases by county and the WNV-positive dead bird variables for the 24 counties with human cases in 2001–2003 is shown in Table 3. The three variables with the highest correlations with human cases were A6 [Positive birds/Tested birds], A7 [(Positive/Tested)*Population] and A8 [(Positive/Tested)*(Population/Area)].

**Table 3:** Correlation matrix, number of human cases with positive and tested dead bird variables^a^

**Pearson Correlation Coefficients, N = 24 (Counties) Prob > |r| under H_0_: Rho = 0**										
	**Pos**	**Humans**	**a1**	**a2**	**a3**	**a4**	**a5**	**a6**	**a7**	**a8**

**Pos**	1.00000									
**Humans**	0.14903	1.00000								
	0.4870									
**a1**	0.91456	0.21947	1.0000							
Pos/area	<.0001	0.3028	0							
**a2**	0.81757	0.22356	0.9614	1.0000						
Tes/Area	<.0001	0.2937	9	0						
			<.0001							
**a3**	0.36780	-0.32477	0.3061	0.2949	1.00000					
pos/pop	0.0770	0.1215	3	5						
			0.1457	0.1618						
**a4**	-	-0.27121	0.0724	0.1586	0.74098	1.00000				
Pos*(pop/area)	0.05282	0.1999	0	7	<.0001					
	0.8064		0.7367	0.4590						
**a5**	0.87792	0.32600	0.9314	0.9098	0.09122	-	1.00000			
(Tested/area)*pop	<.0001	0.1200	2	1	0.6716	0.15399				
			<.0001	<.0001		0.4725				
**a6**	0.62969	0.33804	0.6689	0.5274	0.20231	-	0.53550	1.00000		
Pos/Tested	0.0010	0.1062	8	4	0.3431	0.07312	0.0070			
			0.0004	0.0081		0.7342				
**a7**	0.77953	0.57623	0.8162	0.7401	-	-	0.88204	0.67996	1.00000	
(Pos/Tes)*pop	<.0001	0.0032	1	2	0.11987	0.32893	<.0001	0.0003		
			<.0001	<.0001	0.5769	0.1165				
**a8**	0.28476	0.68430	0.5032	0.5051	-	-	0.60591	0.45424	0.77464	1.00000
(pos/test)*(pop/are a)	0.1774	0.0002	0	9	0.38292	0.28908	0.0017	0.0258	<.0001	
			0.0122	0.0118	0.0648	0.1707				

^a^Each cell provides the correlation coefficient r, and below it the probability value under the null hypothesis r = 0, α = 0.05.

New York State, 2001–2003. Humans = [human WNV cases], Pos = [birds tested positive for WNV], Tested = [birds tested for WNV], A1 = [Positive/Area (county land-area in square miles)], A2 = [Tested/Area], A3 = [Positive/Pop. (county human population, US Census Bureau 2000 estimates)], A4 = [Positive*(Pop./Area)], A5 = [Tested*(Pop./area)],, A6 = [Positive/Tested], A7 = [(Positives/Tested)*Pop.] A8 = [(Positive/Tested)*(Pop./Area)]

These three variables were used in a multiple regression analysis with the number of human cases in a county as the predicted variable. The adjusted R-square for the model using all three variables was 0.3949 (Table 4). As evidenced in the correlation matrix (Table 3), variables A7 and A8 are highly correlated with each other (r = 0.77). Variable A7 can be excluded from the analysis without significant loss of information. The multiple regression model with only variables A6 and A8 yielded a higher adjusted R-square of 0.418 (Table 4). However, the correlation between these two variables is also high (r = 0.454) (Table 3). The multiple regression model with the single variable A8 [(Positive/Tested)*(Pop/Area)] yielded the highest adjusted R-square (Table 4). When repeating the same analysis for predictors with low correlations between each other (Positive, A8 and A3, Table 3), the single variable A8 again yielded the highest adjusted R-square. As a result of this analysis, variable A8 [(Positive/Tested)*(Pop/Area)] was used as the predictor variable for subsequent analyses.

**Table 4:** Multiple regression models for prediction of the number of human WNV cases in a county

Model/Variables	Adjusted R-square
Using three variables most highly correlated with number of human cases	
	
A6, A7, A8	0.395
A6, A8	0.418
A8	0.444
	
Using three variables with lowest intercorrelations	
	
A3, Pos, A8	0.394
A8, A3	0.422
A8	0.444

New York State, 2001–2003. Pos = [birds tested positive for WNV], Tested = [birds tested for WNV], A3 = [Positive/Pop. (county human population, US Census Bureau 2000 estimates)], A6 = [Positive/Tested], A7 = [(Positives/Tested)*Pop.], A8 = [(Positive/Tested)*(Pop./county land-area in square miles)]

### Delay Period

**Figure 1:**
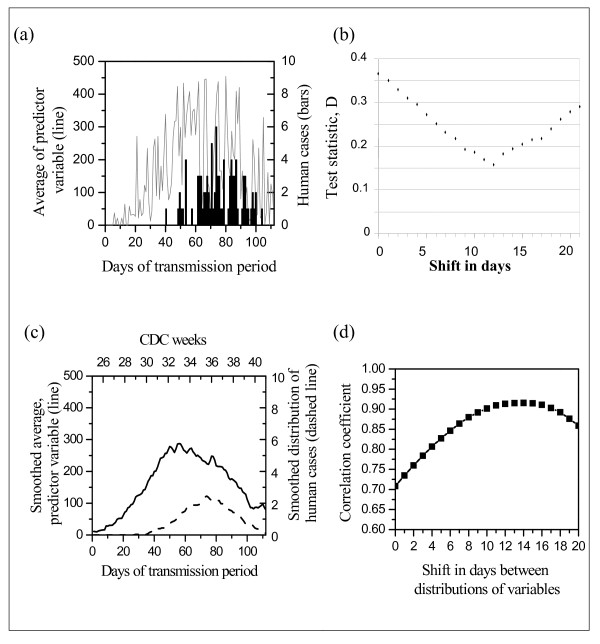
Assessment of delay period between predictor variable [(Positive/Tested)*(Pop./Area)] per county and number of human WNV disease cases. (a) Average of predictor variable (solid curve) versus number of human WNV cases (column bars), by day of transmission period (CDC weeks 26–41). (b) Results of two-sample Kolmogorov-Smirnov test statistic D analysis, for daily distribution of average of predictor variable and number of human cases. (c) Smoothed adjacent point averaging with degree n = 25 points. The smoothed value at day *t *is the average of the data points in the interval [t-(n-1)/2, t+ (n-1)/2], inclusive. Solid curve represents the predictor variable, dashed curve the human cases. (d) Correlation coefficients between smoothed distributions.

Figure 1a shows the daily distributions of means of variable A8 [(Positive birds/Tested birds)*(Population/Area)] and daily distribution of human cases by onset date. Using the Kolmogorov-Smirnov test to determine the best fit (smallest D value) for shift values from 1 to 20 between the daily distribution of the predictor variable and the distribution of human cases, the test identified 12 days as the optimal shift value (Figure 1b).

We also examined the smoothed plot of our two distributions (Figure 1c). We found correlations between human cases at time t and our predictor variable at time t+p. We determined the optimal shift p when the correlation reached its maximum. Although the correlation reached its maximum at 14 days, values for shifts 12, 13, 14 and 15 days were very close to each other, so the optimal shift can be 12–15 days. Figure 1d represents the plot of values of the correlation coefficient for the predictor variable against shifts in days.

### Estimation of WNV disease risk

**Figure 2:**
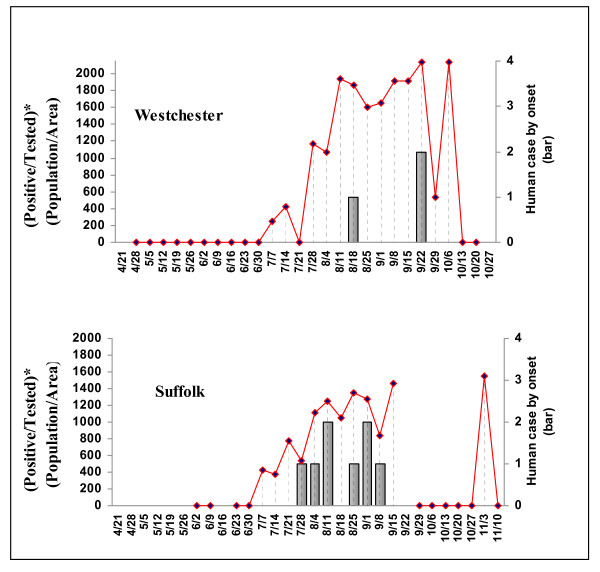
Predictor dead bird variable [(Positive/Tested)*(Population/Area)] and number of human WNV disease cases by week, 2002, Westchester and Suffolk counties, New York.

The weekly value of the predictor variable [(Positive/Tested)*(Population/Area)] was calculated for every county in the analysis, and compared on a timeline with human case onset. Examples for two counties in 2002 are shown in Figure 2.

Based on the previous analysis, we defined the "delay" period as two weeks. Several "signal" values were evaluated for the variable, between 100 and 500. The county was considered exposed if it had a "signal" value at the time of the delay period (two weeks) before the onset of human disease. The results of the CMH test for several signal value thresholds are presented in Table 5. In 2001 and 2002, there were no significant differences in the risk of becoming a WNV case between people who lived in counties with the predictor dead bird variable [(Positive birds/Tested birds)*(Population/Area)] greater than or equal to the "signal" value and the people who lived in counties with [(Positive birds/Tested birds)*(Population/Area)] less than the "signal" value. For 2001, logit estimates of relative risk were used because there were only five human WNV cases and none of them was "unexposed". In 2003 for every threshold considered, the risk of becoming a WNV case among people who lived in counties with the predictor variable greater than or equal to the "signal" value was about four times higher than the risk among people who lived in counties with the predictor variable less than the "signal" value. The highest relative risk was 4.87 for the threshold of 400. For all 3 years combined, the risk of becoming WNV case for people who lived in 'exposed' counties was about two times higher than the risk for people who lived in 'unexposed' counties, regardless of threshold value used.

**Table 5:** Association of weekly predictor variable^a ^values and number of human cases, by signal value

Threshold values	2001 RR^b^ (95%CI)	2002 RR (95% CI)	2003 RR (95% CI)	2001–2003 RR (95% CI)
300	2.79 (0.57, 13.53)	1.43 (0.79, 2.58)	4.03 (1.99, 8.12)	2.21 (1.43, 3.41)
350	2.93(0.92, 13.45)	1.54 (0.79, 2.97)	4.41(2.18, 8.92)	2.34 (1.51, 3.62)
400	3.07(0.63,14.92)	1.48 (0.81,2.69)	4.87 (2.38, 9.90)	2.43 (1.57, 3.77)
450	3.07(0.63,14.92)	1.55 (0.84, 2.84)	4.69(2.33, 9.42)	2.45 (1.58, 3.45)
500	3.07(0.63, 14.92)	1.36 (0.76, 2.42)	4.73 (2.37, 9.46)	2.22 (1.45, 3.40)

^a ^Predictor variable was weekly county values for [(Positive/Tested)*(Population/Area)]

^b ^Relative risks and 95% confidence intervals, CMH test. Due to the small number of human cases in 2001, logit estimates of relative risk were calculated with 0.5 corrections for zero-value cells.

## Discussion

Previous studies have assessed the value of other WNV variables for forecasting potential increases in human disease. In one study that used dead bird surveillance information (WNV positive birds and tested birds) to construct the predictor variables, the study focused on the early transmission season, identified as a six-week period in June-July [21]. Another study utilized dead crow sightings, defining the appropriate period for forecasting as up to two weeks before human case onset based on incubation period, but did not conduct an analysis of delay period [24]. Many studies that tracked WNV-positive birds reported that they were found before onset of human disease. Our study is the first statistical comparison of the daily distributions of the dead bird predictor variable and the number of human cases to confirm a delay period between these two distributions. Using either raw or smoothed data, the delay period was about two weeks, which is consistent with the previous conclusions based on incubation period.

The estimates of relative risk will be affected by the choice of predictor variable. For example, most of the NY human WNV cases in 2001–2003 were reported from counties with a high human population density. Counties with low population density usually had only single WNV cases (Clinton, Orleans, and Yates counties in 2002; Warren, Cattaraugus, Yates, and Dutchess counties in 2003). A notable exception is Broome County in 2002, with seven human WNV cases. Although the overall county human population density in Broome County is relatively low (283.64 persons per square mile), the cases were clustered around the higher population density city of Binghamton. Because the analyses for this study were done at the county level, Broome County was classified as not 'exposed' for the dead bird predictor variable thresholds we considered. This may be one factor affecting the low relative risk estimates for 2002.

To address the issue of the large variation in population density among counties, future studies may benefit from combining data from several states and grouping counties with similar population densities. Different threshold values may be applicable for each group. Another approach would be to assign weights to each county according to its population density, to account for the large variation among counties. The weights can be determined, for example, as a ratio of the population densities of the most populated county to the given county. When the dead bird predictor variable is multiplied by this weight, the counties will become more comparable.

In interpreting these findings, several limitations of the study methodology must be considered. Reporting of dead birds was a passive system, depending solely on the public. Variability in reporting interest between counties could lead to misclassification on the predictor variable, reducing the chance of detecting an association. Not all reported dead birds were collected and tested, which could also affect estimates of the predictor variable. Only reported human cases were used in the analysis, and thus human infection (either asymptomatic or mild, and thus unreported) was likely rarely recognized and reported. This would also reduce the statistical power of detecting an association. The number of NY human cases was relatively small in each year compared to some other states, especially in 2001, which was one reason for conducting our analyses at the county level. With a larger number of cases, analyses could be done at the sub-county level allowing more precise interpretation of risk for vector control decisions. Infection in other counties due to movement of humans and birds will lead to misclassification and reduction of statistical power. Although the relative risks were elevated using the threshold values in the study for all three years, they were only statistically significant for 2003 and all three years combined. More years of data, or combining data across states, would increase the power of our analysis.

Estimation of WNV risk using the CMH test has limitations based on the parameters chosen for this study. This study restricted county entry into the study by having at least 10 birds tested during the transmission season, and the effect of using other numbers of birds for determining inclusion was not examined. Because the predictor variable is [(Positive/Tested)*(Population/Area)], higher levels of birds tested per week could be considered in order to justify that a county has sufficient surveillance to warrant inclusion in the study. Different threshold values could also be considered, and may be appropriate in areas with different WNV disease dynamics and different surveillance systems. The predictor variable consists of two parts: proportion of positive birds and human population density. The proportion can take values between zero and one; therefore the maximum value of our predictor variable is the population density of the particular county. Counties with small population densities may never be able to reach specific threshold values to provide a 'signal' of risk. On the other hand, their small human population size indicates an inherent lower risk of those counties having a human case, in comparison to counties with higher human populations even with the same proportion of infected mosquitoes and birds. Thus, regional rather than county analyses may be more useful in more sparsely populated areas, particularly if such analyses can compare urban and rural areas. However, if using the analyses to determine specific prevention and control activities based on having a "signal" of risk, analyses in smaller geographic units can be more helpful in determining where to target those interventions. Finally, these "signals" were generated based on bird surveillance alone, and it is possible that "signals" developed that also incorporated mosquito surveillance data might offer even better prediction of human case risk, but mosquito surveillance data is often not widely available with rapid test results for real-time analyses.

In New York State, decisions about WNV prevention and control are primarily made at the county level. These decisions have resource consequences, and use of chemicals for mosquito control can raise concerns. Thus, decision-making is usually based on a wide variety of inputs to help determine level of risk and need for control, including more focal information such as dead bird clusters or areas with high mosquito infection rates. Previous studies have indicated the value of dead bird indicators [7-16,21,24], and this study provides a novel statistical approach to demonstrate the value of using WNV-infected dead birds as initial indicator of WNV disease risk for a county. Signals values of our weekly predictor variable above all of the threshold values evaluated in this study were associated with increased risk of WNV disease, with significant associations in one year and across the three years of the study. The North American WNV strain appears to be a phenotype highly virulent to American crows [25]. However, dead bird indicators using American crows may be of less value in the future if there are reductions in the case fatality rate or reduced numbers of birds for surveillance due to WNV die-offs.

## Competing interests

The authors declare that they have no competing interests.

## Authors' contributions

The study was conceived by ME and conducted by AV, under the guidance of IZ. AV developed the manuscript with contributions from ME and IZ. All authors read and approved the final manuscript.
